# Analysis of Serum miRNA in Glioblastoma Patients: CD44-Based Enrichment of Extracellular Vesicles Enhances Specificity for the Prognostic Signature

**DOI:** 10.3390/ijms21197211

**Published:** 2020-09-29

**Authors:** Theophilos Tzaridis, Katrin S Reiners, Johannes Weller, Daniel Bachurski, Niklas Schäfer, Christina Schaub, Michael Hallek, Björn Scheffler, Martin Glas, Ulrich Herrlinger, Stefan Wild, Christoph Coch, Gunther Hartmann

**Affiliations:** 1 Institute of Clinical Chemistry and Clinical Pharmacology, University of Bonn, 53127 Bonn, Germany; theophilos.tzaridis@ukbonn.de (T.T.); ccoch@uni-bonn.de (C.C.); Gunther.Hartmann@uni-bonn.de (G.H.); 2 Division of Clinical Neurooncology, Department of Neurology, Center of Integrated Oncology Aachen-Bonn-Cologne-Düsseldorf, Partner Site Bonn, University Hospital Bonn, 53127 Bonn, Germany; Johannes.weller@ukbonn.de (J.W.); Niklas.Schaefer@ukbonn.de (N.S.); Christina.Schaub@ukbonn.de (C.S.); Ulrich.Herrlinger@ukbonn.de (U.H.); 3 Tumor Initiation & Maintenance Program, NCI-Designated Cancer Center, Sanford Burnham Prebys Medical Discovery Institute, La Jolla, CA 92037, USA; 4 Department I of Internal Medicine, Center for Integrated Oncology Aachen-Bonn-Cologne-Düsseldorf, Partner Site Cologne, CECAD Center of Excellence on ‘‘Cellular Stress Responses in Aging-Associated Diseases’’, Center for Molecular Medicine Cologne, University of Cologne, 50937 Cologne, Germany; Daniel.Bachurski@uk-koeln.de (D.B.); Michael.hallek@uk-koeln.de (M.H.); 5 DKFZ-Division Translational Neurooncology at the West German Cancer Center (WTZ), German Cancer Consortium (DKTK), DKFZ Heidelberg & Partner Site Univ Hospital Essen, 45147 Essen, Germany; b.scheffler@dkfz-heidelberg.de; 6 Division of Clinical Neurooncology, Department of Neurology and West German Cancer Center (WTZ), German Cancer Consortium, University Hospital Essen, 45147 Essen, Germany; martin.glas@uk-essen.de; 7 Miltenyi Biotec & Biomedicine GmbH, 51429 Bergisch Gladbach, Germany; StefanW@Miltenyi.com

**Keywords:** biomarkers, glioblastoma, extracellular vesicles, microRNA, immunoprecipitation, CD44

## Abstract

Glioblastoma is a devastating disease, for which biomarkers allowing a prediction of prognosis are urgently needed. microRNAs have been described as potentially valuable biomarkers in cancer. Here, we studied a panel of microRNAs in extracellular vesicles (EVs) from the serum of glioblastoma patients and evaluated their correlation with the prognosis of these patients. The levels of 15 microRNAs in EVs that were separated by size-exclusion chromatography were studied by quantitative real-time PCR, followed by CD44 immunoprecipitation (SEC + CD44), and compared with those from the total serum of glioblastoma patients (*n* = 55) and healthy volunteers (*n* = 10). Compared to total serum, we found evidence for the enrichment of miR-21-3p and miR-106a-5p and, conversely, lower levels of miR-15b-3p, in SEC + CD44 EVs. miR-15b-3p and miR-21-3p were upregulated in glioblastoma patients compared to healthy subjects. A significant correlation with survival of the patients was found for levels of miR-15b-3p in total serum and miR-15b-3p, miR-21-3p, miR-106a-5p, and miR-328-3p in SEC + CD44 EVs. Combining miR-15b-3p in serum or miR-106a-5p in SEC + CD44 EVs with any one of the other three microRNAs in SEC + CD44 EVs allowed for a prognostic stratification of glioblastoma patients. We have thus identified four microRNAs in glioblastoma patients whose levels, in combination, can predict the prognosis for these patients.

## 1. Introduction

Glioblastomas lacking isocitrate-dehydrogenase (*IDH*) 1 or 2 mutations (IDH-wildtype glioblastomas) are the most aggressive primary brain tumors, mainly occurring in adults, and exhibit a dismal prognosis [1]. First-line treatment includes surgical resection if feasible, followed by radiochemotherapy with temozolomide (TMZ) [2]. Despite a large number of studies on biomarkers for glioblastoma, only one has regularly been applied for prognostic stratification in routine clinical use: Promoter methylation of the *O-6-methylguanine-DNA methyltransferase* (*MGMT*) gene in tumor tissue. Promoter methylation of the *MGMT* gene leads to impaired DNA-repair mechanisms and not only correlates with superior survival, but also leads to a better response to TMZ treatment [3]. Based on this observation, novel data show an enhanced response of combination therapy with TMZ and lomustine (CCNU) for patients with tumors harboring *MGMT*-promoter methylation, and this intensified treatment does not compromise the patients’ quality-of-life [4,5]. Due to the limited number of treatment options for recurrent disease [6], biomarkers capable of stratifying patients are crucial.

MicroRNAs (miRNAs) are non-coding single-stranded RNA molecules with a length of 21–25 nucleotides [7]. After initial processing in the nucleus by RNA polymerases II and III [8], the precursor miRNA (pre-miRNA) is transported into the cytoplasm and then processed by the cytoplasmic RNase III protein Dicer, thereby creating mature miRNA [7,8,9]. The purpose of the mature microRNAs is complex, and not yet fully understood, but usually involves the regulation (mostly repression) of gene expression by binding to either the 3′- or the 5′-untranslated region (UTR) of mRNAs [8,10,11]. microRNAs are loaded into an Argonaute protein (Ago) as part of the RNA-induced silencing complex (RISC) and are thereby protected from cytoplasmic nucleases [7].

The role of microRNAs in glioblastoma has already been extensively examined. They have been shown to be involved in invasiveness and proliferation (e.g., hsa-miR-21 or hsa-miR-15b) [12], to induce angiogenesis [13], and to modulate the innate immune system by polarizing it towards M2 macrophages and thereby inducing an immunosuppressive environment (hsa-miR-21 and hsa-miR-451) [13]. Interestingly, many of these functions require the trafficking of miRNA between cells, which occurs through either gap junctions or the exchange of extracellular vesicles (EVs) [13,14]. While some miRNAs are mainly found in miRNA-binding protein complexes, such as Ago-2, or high-density lipoproteins (HDL), other types of miRNAs are uniquely packaged into EVs [15], which makes them a potentially interesting biomarker in tumor diagnostics [16].

EVs are small particles formed by either direct budding of the plasma membrane (typically “large EVs”) or by the fusion of a multivesicular endosome with the plasma membrane (typically “small EVs”), thus releasing the EVs into the extracellular space [17]. In a recent study, we identified relevant protein markers that are highly elevated in EVs from the serum of patients with glioblastoma, compared to those from healthy volunteers (HV), and these proteins are capable of detecting tumor progression [18]. Many studies have identified microRNAs in the biofluids (e.g., cerebrospinal fluid (CSF), serum and plasma) of patients with glioblastoma, which can be EV-independent [19] or contained in EVs [20]. While some reports have demonstrated the upregulation of microRNAs in patients with high-grade glioma compared to low-grade glioma [21], no report has thus far shown the potential of microRNAs in biofluids to stratify patients into different prognostic groups at critical time-points during treatment. Moreover, it is unknown whether the detection of any relevant microRNAs in the serum of glioma patients can be enhanced through specifically capturing tumor-derived EVs.

In this study, we evaluated suitable methods for the separation of EVs that could enrich the potentially glioblastoma-relevant microRNAs within the samples. In addition, we tested whether these microRNAs could serve as biomarkers to determine patient prognosis when purified from serum EVs (EV microRNAs) or as microRNAs directly from total serum (total serum microRNAs).

## 2. Results

### 2.1. Enrichment of Disease-Relevant microRNAs in Serum EV Samples Using Size-Exclusion Chromatography (SEC) Followed by CD44-Based Immunoprecipitation

**Figure 1:**
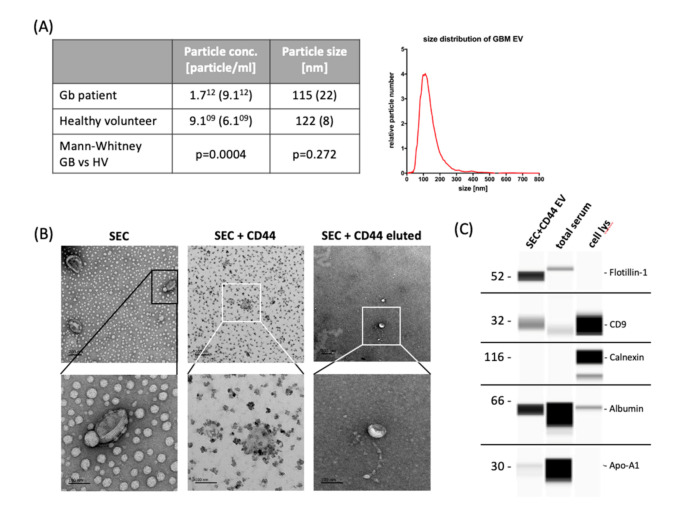
Yield and characteristics of serum-derived extracellular vesicles. (**A**) NTA of extracellular vesicles (EVs) isolated from 500 µL serum by size-exclusion chromatography (SEC) to determine the yield and particle size. The table states the mean particle concentration and mean modal size of 55 glioblastoma patients and 5 healthy volunteers (HV). Standard deviation is given in parentheses; histograms: exemplary size distribution of glioblastoma-EVs measured by NTA. (**B**) Transmission electron microscopy images showing the typical EV morphology. Scale bar represents, in all images, 200 nm in the upper row (30k × magnification) and 100 nm in the lower row (110k × magnification). Images in the left lane show EVs after SEC, middle lane images show CD44-captured SEC-EVs, and right lane images represent EVs that were diluted from CD44-beads. (**C**) Wes ProteinSimple immunodetection confirming the presence of the EV-markers flotillin-1 and CD9 and the absence of non-EV protein calnexin in SEC + CD44 EV preparations. Cell and total serum lysates were used as positive controls for the non-EV marker calnexin, apolipoprotein A1, and serum albumin, respectively. NTA = nanoparticle tracking analysis; cell lys = cell lysate of PBMCs (1:10) or glioblastoma cell line Gli36 (1:100); Apo-A1 = apolipoprotein A1.

Our study included 55 patients in total, 26 (47.3%) of which were treated within the multicenter Phase III CeTeG/NOA-09 trial [4] and 29 (52.7%) in the Division of Clinical Neurooncology of the University Hospital of Bonn. The median age at diagnosis was 56 years (range: 19–77 years) and the median overall survival was 2.35 years (range: 0.39–5.17 years). The vast majority of patients (53/55, i.e., 96.4%) had *IDH*-wildtype tumors and *MGMT* promoter methylation was observed in 33 (60%) cases (Table S1). Sampling was performed at the Q3 time point (i.e., the third quarter of the first year after diagnosis, 6–9 months after the initiation of primary radiotherapy and chemotherapy) for 54/55 patients and in the fifth month for one patient. Consistent with previous results [21], serum from glioblastoma patients showed a much higher concentration of small EVs (size range: 87–166 nm) compared to HV when they were isolated by using size-exclusion chromatography (SEC, Figure 1A). In order to specifically isolate relevant glioblastoma-associated EVs, we subsequently performed immunoprecipitation using CD44 (SEC + CD44), which we had previously identified in a screen of serum EV proteins as being important in tumor progression [18]. Using this technique, we were able to capture a distinct EV subpopulation, as shown in Figure 1. Immunodetection was used to determine the presence of EV marker proteins flotillin-1 and CD9. Calnexin, which is a protein associated with the endoplasmic reticulum, serum protein albumin and apolipoprotein-A1 were absent or strongly reduced after EV isolation via SEC + CD44 (Figure 1C). Transmission electron microscopy (TEM) analysis showed that the CD44-enriched fraction mainly contained EVs with a size and shape typical of small EVs (Figure 1B). The TEM images show that lipoprotein contamination present after SEC (Figure 1B, left lane) is diminished after additional immunoprecipitation with CD44 beads (Figure 1B, middle lane). To allow the quantification of SEC + CD44 EVs, they were effectively diluted from capture-beads (visible as small black dots in middle lane pictures), as shown in Figure 1B, in the right lane.

**Figure 2:**
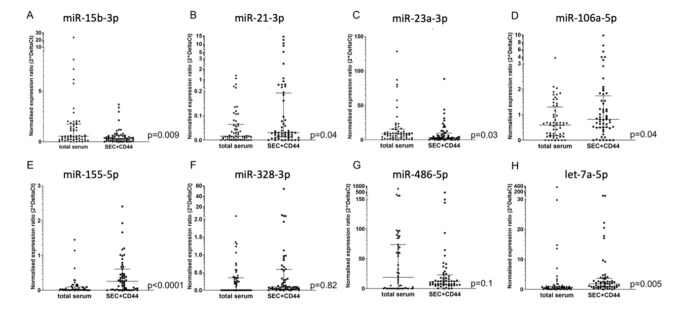
Comparison of microRNA detection in glioblastoma serum, depending on the source. (**A**) miR-15b-3p, (**B**) miR21-3p, (**C**) miR-23a-3p, (**D**) miR-106-5p, (**E**) miR-155-5p, (**F**) miR-328-3p, (**G**) miR-486-5p, (**H**) let-7a-5p. Depicted is the normalized expression ratio (2^deltaCt) of eight microRNAs (**A**–**H**) in total serum and in SEC + CD44-purified EVs from the serum of glioblastoma patients (*n* = 55). Expression levels were compared using a Wilcoxon rank sum test. Note that miR-15b-3p shows higher levels in total serum, as opposed to miR-21-3p and miR-106a-5p, which are upregulated in SEC + CD44 EVs. For miR-328-3p, no consistent up- or downregulation was observed between the two samples, which was partially because only 26/55 (47%) total serum samples were above the limit of detection. Bars depict the median value and the interquartile range.

Based on extensive screening of the literature, we selected 15 microRNAs that have been described to be present and relevant in the serum and plasma of glioblastoma patients (Table S2). Out of these 15 pre-screened microRNAs, we identified eight that consistently had Ct values lower than 36 in our glioblastoma patients. To determine if these miRNAs are more abundant in CD44-enriched EVs (SEC + CD44 EVs) compared to miRNAs isolated from total serum, we studied the levels of these microRNAs in the serum of glioblastoma patients (*n* = 55) at the Q3 time point. In total, we detected an enrichment of five out of the eight microRNAs tested in SEC + CD44-purified EVs compared to total serum (miR-21-3p, miR-106a-5p, miR-155-5p, and let-7a-5p with significantly higher levels and miR-486-5p with a non-significant trend toward a higher level, Figure 2A–H). Two out of eight miRNAs showed significantly higher levels in total serum compared to the purified EVs (miR-15b-3p and miR-23a-3p, Figure 2), indicating that these miRNAs are not enriched in SEC + CD44 EVs.

**Figure 3:**
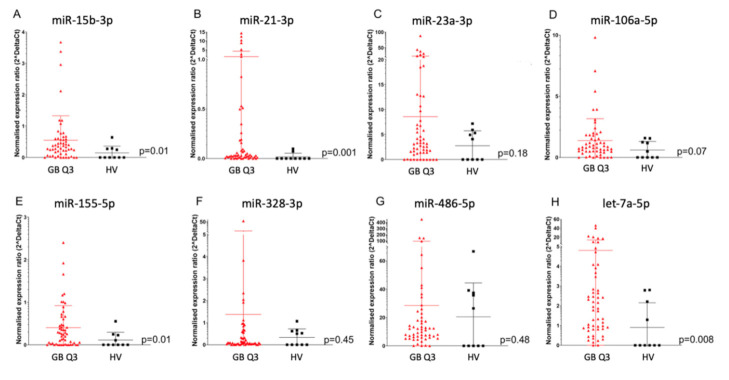
MicroRNA expression in SEC + CD44 EV. Normalized expression ratio (2^deltaCt) of eight microRNAs (**A**–**H**) in SEC + CD44 EV from glioblastoma patients (*n* = 55) versus HV (*n* = 10, average of two time points). (**A**) miR-15b-3p, (**B**) miR21-3p, (**C**) miR-23a-3p, (**D**) miR-106-5p, (**E**) miR-155-5p, (**F**) miR-328-3p, (**G**) miR-486-5p, (**H**) let-7a-5p. Expression levels were compared using the Mann–Whitney U test. Bars depict the median value and the interquartile range.

When comparing SEC + CD44-purified EV microRNA from the serum of glioblastoma patients to HV, we saw significantly higher levels of miR-15b-3p, miR-21-3p, miR-155-5p, and let-7a-5p in glioblastoma patients (*p* = 0.01, *p* = 0.001, *p* = 0.01, and *p* = 0.008, respectively) and a non-significant trend for miR-23a-3p and miR-106a-5p (*p* = 0.18 and *p* = 0.07, respectively), while no significant difference was detected for miR-23a-3p, miR-328-3p, and miR-486-5p (*p* = 0.45 and *p* = 0.48 respectively, Figure 3A–H).

### 2.2. Four SEC+CD44-EV miRNAs and Total Serum miR-15b-3p Correlate with Survival in Glioblastoma Patients

To evaluate the prognostic potential of different microRNAs, we correlated the normalized expression ratio with the overall survival of glioblastoma patients (*n* = 55). miR-15b-3p, miR-21-3p, miR-106a-5p, and miR-328-3p in SEC + CD44 EVs showed a significant correlation with survival, with miR-15b-3p, miR-21-3p, and miR-328-3p exhibiting a negative correlation (high levels were associated with an inferior prognosis) and miR-106a-5p a positive correlation (high levels were associated with a better prognosis; Table 1A). While miR-15b-3p showed a weak correlation (Spearman r = −0.27), miR-21-3p, miR-106a-5p, and miR-328-3p showed intermediate correlation values (absolute Spearman r > 0.34). Interestingly, when considering total serum microRNAs, only miR-15b-3p correlated with inferior survival (Spearman r = 0.4) and miR-328-3p was not measurable in 29/55 (53%) patients (Table 1B). For all of the other microRNAs analyzed, no correlation with survival was found in either SEC + CD44 EVs or total serum (data not shown).

Interestingly, we found distinct differences of miR levels in SEC + CD44 EVs, depending on the MGMT promoter methylation status, with miR-15b-3p, miR-21-3p, and miR328-3p being enriched in MGMT-non-methylated and miR-106a-5p in MGMT-methylated patient samples (Figure S1). For total serum, we saw higher levels of miR-15b-3p in MGMT-non-methylated compared to MGMT-methylated patient samples (Figure S2). When only assessing the prognostic potential of the above-mentioned markers for MGMT-methylated patients, we observed a non-significant correlation with overall survival for miR-21-3p and miR-328-3p in SEC + CD44 EVs (data not shown).

**Figure 4:**
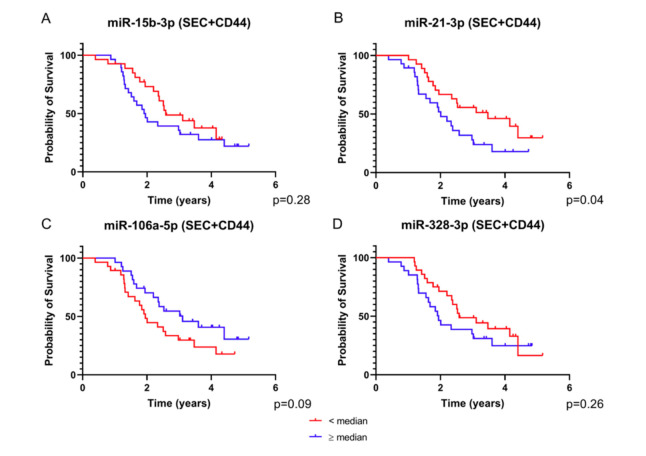
Survival analysis based on the expression of SEC + CD44 EV microRNA. Depicted are survival curves using a dichotomous assessment based on microRNA levels of SEC + CD44 EVs. ((**A**) miR-15b-3p, (**B**) miR-21-3p, (**C**) miR-106a-5p, (**D**) miR-328-3p) from glioblastoma patient serum (*n* = 55). Red color indicates patients with values equal to or higher than the median and blue color represents values lower than the median normalized expression ratio. Note that all four microRNAs were able to stratify the patients into different prognostic subgroups, albeit not reaching statistical significance. The p value for miR-21-3p is above the critical Benjamini–Hochberg value (0.03, Tables S3 and S4). Also note that for miR-106a-5p, higher values correlate with a better prognosis.

The four microRNAs that were identified as putative prognostic markers were selected for dichotomous assessment based on the median of the normalized expression ratios and using Kaplan–Meier curves for data visualization. To define the subgroups, we calculated the median value of the normalized expression for each microRNA and split the patients into two groups, with one group including patients with expression values lower than the median and the other group with values equal to or higher than the median. In an analysis of single SEC + CD44 EV microRNAs, we saw a curve separation for all four miRNAs, which was less pronounced for miR-15b-3p and miR-328-3p (Figure 4A–D). Consistent with our correlation data, the subgroup of patients with levels of miR-106a-5p higher than the median had a clear survival benefit compared to those with lower levels, as opposed to miR-21-3p, where high levels of the miRNA defined a prognostically inferior group (Figure 4B,C).

**Figure 5:**
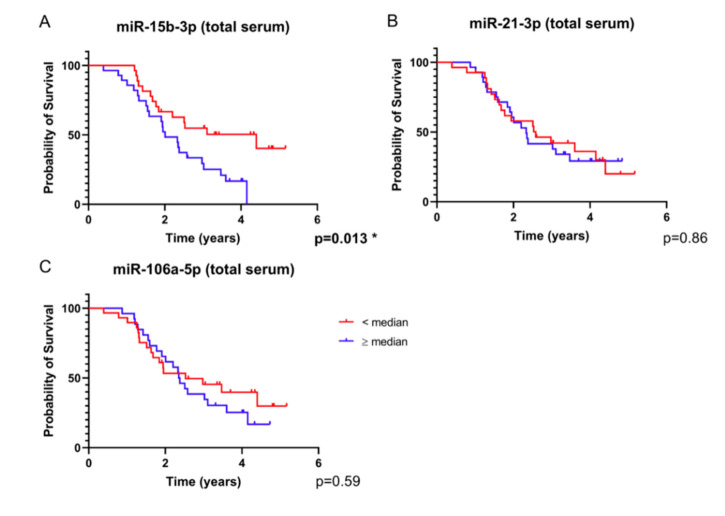
Survival analysis based on total serum microRNA expression. Depicted are survival curves using a dichotomous assessment based on microRNA levels ((**A**) miR-15b-3p, (**B**) miR-21-3p, (**C**) miR-106a-5p) from total serum of glioblastoma patients (*n* = 55). Red color indicates patients with values equal to or higher than the median and blue color represents values lower than the median normalized expression ratio. For miR-328-3p, no graph was generated because more than 50% of the values were non-measurable. Note that the separation of the curves based on miR-15b-3p is significant after Benjamini–Hochberg correction for multiple testing (Tables S3 and S4).

In analyses of single total serum microRNAs, we observed a clear curve separation for miR-15b-3p, as opposed to miR-21-3p and miR-106a-5p (Figure 5A–C). For miR-328-3p, no Kaplan–Meier curve was generated, because more than 50% of the values were below the detection limit.

### 2.3. Combination of SEC + CD44-EV- and Total Serum-microRNAs Allows Patient Stratification into Prognostically Relevant Subgroups

**Figure 6:**
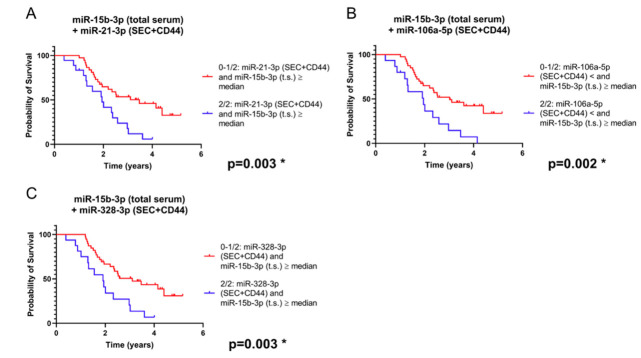
Survival analysis based on a combination of microRNA levels in total serum (t.s.) and/or SEC + CD44 EVs (combinations: miR-15b-3p in t.s. plus (**A**): miR-21-3p in SEC + CD44 EVs; (**B**): miR-106a-5p in SEC + CD44 EVs; (**C**): miR-328-3p in SEC + CD44 EVs). Depicted are survival curves using a dichotomous assessment from glioblastoma patients (*n* = 55) in both SEC + CD44 separated serum EVs and total serum. Blue color indicates patients whose microRNA profile fulfils both conditions, while red color indicates patients whose profile fulfils at most one out of two conditions. Note that the combination of miR-15b-3p in serum with each of these four microRNAs in SEC + CD44 EVs allowed for a prognostic stratification of this patient population, even after Benjamini–Hochberg correction (Table S1).

We next sought to determine whether a combination of different microRNAs results allows a more precise survival prediction compared to single microRNA analysis. Indeed, when we combined the analysis of miR-15b-3p in total serum with each of the other three EV-contained microRNAs, the prediction of prognosis was improved (based on the *p*-value determined) compared to the single microRNA analyses (Figure 6A–C).

**Figure 7:**
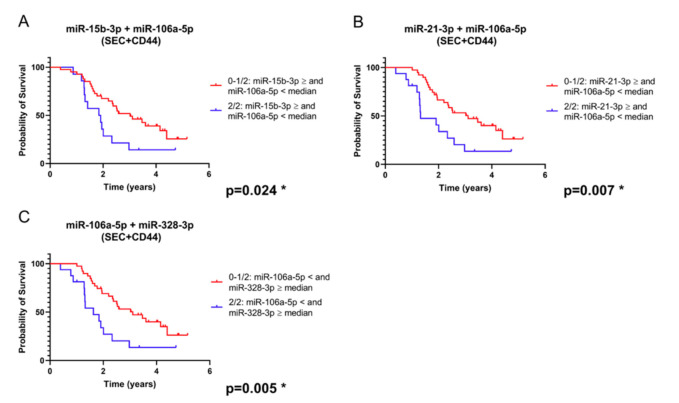
Survival analysis based on the expression of a combination of microRNAs isolated from SEC + CD44 EV (combinations: miR-106a-5p plus (**A**): miR-15b-3p; (**B**): miR-21-3p in SEC + CD44 EVs; (**C**): miR-328-3p). Depicted are survival curves using a dichotomous assessment based on a combination of microRNA levels from glioblastoma patients (*n* = 55) in SEC + CD44 separated serum EVs. Blue color indicates patients whose microRNA profile fulfils both conditions, while red color indicates patients whose profile fulfils at most one out of two conditions. Note that the combination of miR-106a-5p with each of the other three microRNAs allowed for a prognostic stratification of this patient population, even after Benjamini–Hochberg correction for multiple testing (Tables S3 and S4).

When combining markers tested only in SEC + CD44 EVs, three of these combinations, always including miR-106a-5p as one of the two microRNAs, yielded prognostically significant subgroups (Figure 7A–C). Because comparing multiple variables can give a bias and lead to a high risk of false significant results (type I error), we applied Benjamini–Hochberg correction [22]. Importantly, the combinations of microRNAs in Figure 6 and Figure 7, as well as miR-15b-3p in total serum (Figure 5A), remained significant after this correction, as opposed to a single analysis of miR-21-3p (Figure 4B).

## 3. Discussion

In this study, we report on a new method for EV separation using the already established method of size-exclusion chromatography (by using qEV columns from IZON^®^), followed by immunoprecipitation with CD44-conjugated beads, thereby allowing specific enrichment of glioblastoma-associated EVs from patient serum. Using this novel method for EV separation, as well as total serum analyses, we identified a panel of four miRNAs suitable as biomarkers (alone or in combination) that allow for the prognostic stratification of glioblastoma patients at a relevant time point of the disease course.

Glioblastoma is a devastating disease which is currently lacking established biomarkers to aid in the prediction of prognosis and treatment decisions. Although progression-free survival (PFS) has been discussed as a surrogate parameter for overall survival in clinical trials of glioblastoma [23], it is mainly based on radiological findings [24,25], which are known to yield equivocal results [26]. Therefore, identifying biomarkers which would predict prognosis at an early time point during treatment is crucial. The median PFS is heterogeneous, and frequently ranges from 6 to 8 months [27,28]. In this study, we therefore chose the third quartile (6–9 months after the start of the adjuvant treatment) as a time for measuring microRNAs in serum and serum-derived EVs. Robust and feasible prognostic biomarkers at this critical time-point could therefore be highly relevant for the course of disease and help both patients and physicians in developing treatment strategies.

We have previously identified a key role for CD44, found on the surface of serum-EVs from glioblastoma patients, in the detection of tumor progression [18]. Due to a high upregulation of CD44 in glioblastoma patients compared to HV, we hypothesized that the immunoprecipitation of EVs with CD44 would lead to an enrichment of glioblastoma-specific EVs. The rationale for first separating the EVs using SEC was to ensure a purer sample of CD44-carrying EVs and minimize the risk of capturing EV-independent CD44, which is known to be present on various cell types, including T-cells and macrophages [29]. Furthermore, we intended to reduce the amount of soluble CD44 in the sample, which is shed by matrix-metalloproteases from the surface of tumor and non-tumor cells [30]. CD44 can also be found on EVs from different non-malignant cells, such as B- or T-cells [31,32], thus, we cannot exclude the possibility that some non-glioblastoma EVs are found in our SEC + CD44 EV fractions. However, the analysis of SEC-enriched serum-derived EVs from HV showed significantly lower CD44 levels compared to EVs from the serum of glioblastoma patients, indicating that CD44-positive EVs are enriched and highly relevant in glioblastoma patients [18]. This is in line with the intriguing hypothesis that glioblastoma is actually a systemic disease, as glioblastoma cells are known to affect non-tumor cells [33], suggesting that the EV-composition of glioblastoma patients differs from that of HV and thus could serve as a biomarker, irrespective of their origin.

The microRNAs selected for this project have all previously been studied in glioblastoma, either in total serum or EVs [19,20,34,35,36]. While some of these papers report that the majority of extracellular microRNAs are found bound to Ago [14], other studies claim that the majority of extracellular microRNAs are found inside small or large EVs [15,37,38]. Either way, it is clear that EV-associated microRNAs play a central role in intercellular communication and are capable of regulating key oncogenic processes, such as metastasis formation [16,39]. Ebrahimkhani et al. performed deep sequencing of exosomal microRNAs and discovered a panel of microRNAs that were not only upregulated in EVs from glioma patients, but were also capable of discriminating between *IDH*-mutated glioma and *IDH*-wildtype glioblastoma [34]. To account for differences between microRNAs found in total serum and glioblastoma-associated EVs, we examined the microRNA levels in both states and found a significant upregulation in four out of eight microRNAs, thus supporting our rationale for an enrichment of the relevant microRNAs through this isolation technique. Intriguingly, miR-328-3p, which was one of the microRNAs implicated in Ebrahimkhani et al. [34], did not show an increased concentration in EVs, yet its levels correlated with survival in our glioblastoma cohort. This finding underlines the importance of biological relevance rather than absolute quantitative changes in biomarker concentrations. miR-23, albeit having no prognostic significance in our study, might exude biological relevance based on its relatively strong increase in glioblastoma EVs. On the other hand, miR-15b-3p was found at higher levels in total serum and possessed the highest prognostic relevance using single microRNA analysis, while allowing for an even better stratification when combined with other microRNAs in SEC + CD44 EVs. Therefore, our data suggest that both targeted EV and total serum microRNAs should be studied for assessing the prognosis of glioblastoma patients. Notably, this is, to the best of our knowledge, the first study highlighting the prognostic potential of these biomarkers for glioblastoma patients.

The assessment of microRNA levels based on the MGMT methylation status showed that microRNAs indicating a non-favorable outcome (miR-15b-3p, miR-21-3p, and miR328-3p) were elevated in MGMT-non-methylated patient samples, while miR106a-5p showed lower levels compared to MGMT-methylated samples, thereby possibly highlighting a correlation of microRNAs with biologically aggressive tumors. While we only saw non-significant trends towards inferior survival for MGMT-methylated patients showing higher levels of miR-21-3p and miR-328-3p (data not shown), caution is warranted due to the low patient numbers.

All of the microRNAs from our prognostic panel have also been shown to carry important biological functions in glioblastoma, although their up- or downregulation in glioblastoma has not been conclusively resolved. miR-21-3p in EVs secreted by glioblastoma cells is known to promote oncogenesis, angiogenesis, and microglia activation [13] and has unanimously been described to be upregulated in the plasma of glioma patients [40], which is consistent with our data showing higher levels of miR-21-3p in glioblastoma-EVs compared to HV, as well as prognostic significance in SEC + CD44-purified EVs. The levels of miR-15b have also been shown to correlate with a high proliferation of glioma cells in vitro [12], but the levels in the serum of glioma patients were lower compared to HV [41]. Nevertheless, higher serum levels of miR-15b correlated with a higher WHO grade, thereby indicating that more aggressive tumors exhibit higher ratios of this microRNA compared to less proliferative tumors, which corresponds to the survival data in our study. Contradictory data have been published regarding the roles of miR-106a and miR-328 in glioma. High levels of miR-106a in glioma cells have been associated with reduced proliferation and increased apoptosis [12], but in other publications, have been associated with invasiveness [42]. Zhi and colleagues identified Fas-activated serine/threonine kinase (FASTK) as a direct target of miR-106a-5p and showed that a reduced expression of miR-106a-5p or increased expression of FASTK is significantly associated with poor survival in human astrocytoma patients [43], which is compatible with our data. While miR-328 has been shown to mediate the invasiveness of glioma cells via the downregulation of *Secreted Frizzled-related protein 1* (*SFRP1*) [44], Ebrahimkhani et al. reported that serum EVs from glioblastoma patients exhibited lower levels of miR-328 compared to HV [34]. Notably, the EVs used in this study were non-specifically purified from whole serum. Therefore, it would be tempting to speculate that a more specific separation of EVs using CD44 as a target antigen, and thereby enriching the population for glioblastoma EVs, would lead to a correlation of high miR-328 levels with a lower prognosis, as was the case in our study and as reported by Delic et al. [44].

In this study, we used serum as the biofluid source for miRNAs. While plasma used to be the preferred biomaterial for conducting EV studies, because coagulation activates platelets, resulting in an increased release of platelet-derived EVs [45], EVs and microRNAs are now increasingly being evaluated from the serum of glioblastoma patients [19,34]. Intriguingly, large-scale studies of different RNA subclasses, including microRNAs, in biofluids revealed only minor differences in the concentrations between serum and plasma [46,47]. These differences increased substantially if different RNA-extraction and EV-separation methods were applied, implying that both biofluids could yield similar results when established and validated protocols are used for purification. In our study, we used the miRNeasy kit by Qiagen for RNA extraction, as this method was identified as a suitable extraction method for high-yield and high-quality exosomal RNA [47]. There is still debate over what is the most suitable reference microRNA for calculating deltaCt. While some studies have used raw Ct values or exogenous non-human microRNAs as a reference, newer studies have discouraged the use of these conventions and instead recommend the use of at least one, and ideally two, microRNAs as a reference [48]. Based on these findings, we had previously performed screening for suitable reference microRNAs and identified miR-103 and miR-484 as two reliable housekeeper miRNAs (data not shown).

This is, to the best of our knowledge, the first study defining a microRNA signature (in EVs and cell-depleted serum) that shows a direct correlation with the survival of glioblastoma patients, thereby allowing prognostic stratification. Moreover, we introduce a novel approach for separating and enriching glioblastoma-specific EVs by combining size-exclusion chromatography and immunoprecipitation with CD44 as a unique target antigen. This optimized enrichment of glioblastoma-specific EVs leads to a more precise and sensitive prediction of prognosis compared to an unspecific serum-derived microRNA analysis. These encouraging data remain to be confirmed in larger prospective clinical trials, which could then pave the way for the introduction of an miRNA biomarker signature for clinicians, using methods that are feasible and time-efficient for routine diagnostic laboratories.

## 4. Materials and Methods

### 4.1. Ethical Approval

Studies on two cohorts of glioblastoma patients were separately approved by the Ethical Committee of the University of Bonn (protocol number for patients treated in Bonn: 182/08 and in the CeTeG/NOA-09 trial: 093/10) and on HV (Protocol number: 007/17).

### 4.2. Sample Collection

Serum was collected in 9 mL serum (S-Monovette, Sarstedt, Nuembrecht, Germany) tubes from HV and glioblastoma patients. For glioblastoma patients, blood was drawn at the time of an MRI visit in the third quartile period of their adjuvant treatment (i.e., 6–9 months after the initial diagnosis). For HV, blood was drawn at two different time-points with a time interval of three months. After a resting period of 30 min at room temperature (RT), the samples were centrifuged for 15 min at 2000× *g* at RT, followed by a further centrifugation step for 20 min at 3000× *g* at 6 °C. After filtration with a 0.45 µm filter, the serum was stored in aliquots at −80 °C.

### 4.3. EV Separation

EVs from serum samples were separated by size-exclusion chromatography (SEC) using the sepharose-based qEV columns (iZON Science, Christchurch, New Zealand), according to the manufacturer’s recommendations. In short, 0.5 mL of serum was applied to the column and the EVs were eluted with Hank’s balanced salt solution (HBSS). Next, 500 µL fractions were collected, fractions 8 to 10 were pooled, and a protease inhibitor (cOmplete, EDTA-free Protease Inhibitor Cocktail, Roche, Mannheim, Germany) was added to a final one-fold dilution. Subsequently, the combined fractions 8–10 (in total, 1.5 mL) were used for immunoprecipitation with 50 µL CD44-conjugated MicroBeads (CD44: Clone DB105) that were specifically designed for EV isolation and produced for this study. Separation of the EV-bound MicroBeads was performed with µColumns using the µMACS separator, according to the manufacturer’s protocol (all Miltenyi Biotec, Bergisch Gladbach, Germany). EVs were eluted in 110 µL HBSS (ThermoFisher Scientific, Waltham, MA, USA) with protease inhibitor (Roche, Mannheim, Germany).

### 4.4. Nanoparticle Tracking Analysis (NTA)

As previously described, ZetaView Nanoparticle Tracking (Particle Metrix, Meerbusch, Germany) was used for NTA, according to the manufacturer’s guidelines [49].

### 4.5. Transmission Electron Microscopy (TEM)

TEM was conducted based on the protocol previously described by Bachurski et al. [49]. Briefly, after loading 5 µL of an EV sample onto formvar-coated copper grids (Science Services, Munich, Germany), the EVs were fixed with 2% paraformaldehyde for 5 min, washed with PBS, fixed again for 5 min with 1% glutaraldehyde, washed with ddH_2_O, and incubated with contrast dye (1.5% uranyl acetate) for 4 min. Images were captured with a Gatan OneView 4K camera (Gatan, Pleasanton, CA, USA) on a Jem-2100Plus microscope (JEOL) operating at 200 kV.

### 4.6. Wes^TM^ Simple Immunodetection

The presence of the EV markers CD9 and flotillin-1 and the absence of the endoplasmic-reticulum protein calnexin in the purified EV samples were confirmed using Wes^TM^ Simple Western technology with the Wes instrument (ProteinSimple, San Jose, CA, USA). In this study, 3 µL EVs were combined with 1 µL 0.1 × sample buffer and 1 µL 5 × fluorescent master mix for each lane. Analyses for flotillin-1 (clone 18/flotillin-1; BD Biosciences, Dilution: 1:100), calnexin (clone: C5C9; Cell Signaling Technology, Dilution: 1:80), apolipoprotein A1 (polylonal, R&D Systems, Concentration: 5 µg/mL), and human serum albumin polylonal (R&D Systems, Concentration: 5 µg/mL) were conducted under reducing (DTT-based buffer) conditions, while CD9 (clone D801A, Cell Signaling Technology, Dilution: 1:80) analysis was run under non-reducing conditions and using the 12–230 kDa Wes Separation Module. Anti-rabbit, anti-mouse, and anti-goat antibody detection modules (all from ProteinSimple) were used, according to the manufacturer’s instructions. The default run conditions were changed to stocking-gel uptake: 22 s; sample uptake: 15 s; primary-antibody: 90 min; and secondary-antibody: 40 min incubation. Data analysis was performed with Compass software (ProteinSimple).

### 4.7. RNA Extraction and Quantitative Real-Time Polymerase Chain Reaction (qRT-PCR)

RNA extraction was performed using the Qiagen Micro-RNeasy kit, according to the manufacturer’s protocol (Qiagen, Hilden, Germany). Following RNA extraction, equal volumes of the RNA samples were used for cDNA synthesis using a TaqMan Advanced miRNA cDNA Synthesis Kit, according to the manufacturer’s protocol (ThermoFisher Scientific, Waltham, MA, USA). qRT-PCR was performed by using a QuantStudio 7 with TaqMan Advanced Control miRNA Assay (Table S2, both ThermoFisher Scientific, Waltham, MA, USA). miR-103a-3p and miR-484 were used as reference miRNAs (Table S2). The QuantStudio 7 PCR protocol included (1) enzyme activation at 95 °C for 20 s and (2) 40 cycles of denaturation (1 s at 95 °C) and annealing/extension (20 s at 60 °C).

### 4.8. Data Deposition

We have submitted all relevant data from our experiments to the EV-TRACK knowledgebase (EV-TRACK ID: 200051) [50].

### 4.9. Statistical Analysis

Statistical analysis was performed using GraphPad Prism software (Version 8.2.1, La Jolla, CA, USA). A Mann–Whitney *U* test was used to detect differences in the EV concentration, as well as differences in microRNAs between glioblastoma patients and HV. To compare levels of microRNAs isolated from total serum and SEC + CD44 EVs, a Wilcoxon signed-rank test was applied. Survival curves were generated using Kaplan–Meier plots and groups were evaluated using a log-rank test. The correlation of microRNA levels with survival was investigated using a nonparametric Spearman’s rank correlation coefficient.

Since multiple microRNAs were tested, we corrected for multiple testing using the Benjamini–Hochberg procedure [22]. Out of the measured microRNAs used for dichotomous survival analysis, four out of four were measurable in SEC + CD44, but only three out of four in cell-depleted serum (miR-15b-3p, miR-21-3p, and miR-106a-5p, but not miR-328-3p). Therefore, we included seven groups in the single analysis and 21 in the two-fold combination analysis (Tables S3 and S4), leading to a total of m = 28 groups. After ranking the *p*-values (value i, range 1–28) and defining a false discovery rate (FDR = q) of 10%, we calculated the Benjamini–Hochberg critical value q*(i/m). If the log-rank *p* value was below the Benjamini–Hochberg critical value, the *p* values were considered statistically significant (*).

## 5. Conclusions

A panel of four microRNAs (miR-15b-3p, miR-21-3p, miR-106a-5p, and miR-328-3p) isolated from extracellular vesicles that were purified using size-exclusion chromatography and CD44-based immunoprecipitation in combination with total serum analysis allowed us to predict the prognosis of glioblastoma patients. Further analyses in larger prospective clinical trials are still warranted before these novel biomarkers become established in routine clinical practice.

## Figures and Tables

**Table 1:** Correlation of the survival of 55 patients with microRNA levels from SEC + CD44 EV (**A**) or total serum (**B**) using the Spearman’s rank correlation coefficient, r. Depicted are the Spearman r values showing either a positive or negative correlation, the *p* value, and the degree of significance (* *p* < 0.05, ** *p* < 0.01, *** *p* < 0.001, n.s. = not significant). For miR-328-3p in total serum, no correlation with survival was calculated because 53% of the values were non-measurable.

(A)				
SEC + CD44	miR15b-3p	miR-21-3p	miR-328-3p	miR-106-5p
Spearman R	−0.2746	−0.4372	−0.3407	0.3501
*p* (two-tailed)	0.043	0.00008	0.011	0.009
*p* value	*	***	*	**
**(B)**				
**Total serum**	**miR15b-3p**	**miR-21-3p**	**miR-328-3p**	**miR-106-5p**
Spearman R	-0.3947	0.0225	-	0.06753
*p* (two-tailed)	0.003	0. 78	-	0.62
*p* value	**	n.s.	-	n.s.

## Supplementary Materials

The following are available online at https://www.mdpi.com/1422-0067/21/19/7211/s1.

## Abbreviations

MDPI	Multidisciplinary Digital Publishing Institute
LD	linear dichroism
EV	extracellular vesicles
HV	healthy volunteers
SEC	size-exclusion chromatography
