# Supplementary material for: Analysis of Serum miRNA in Glioblastoma Patients: CD44-Based Enrichment of Extracellular Vesicles Enhances Specificity for the Prognostic Signature
Source: Int J Mol Sci. 2020 Sep 29;21(19):7211. doi: 10.3390/ijms21197211 (PMC7583802; doi:10.3390/ijms21197211)
Supplement: Supplementary file 1 [file ijms-21-07211-s001.pdf]

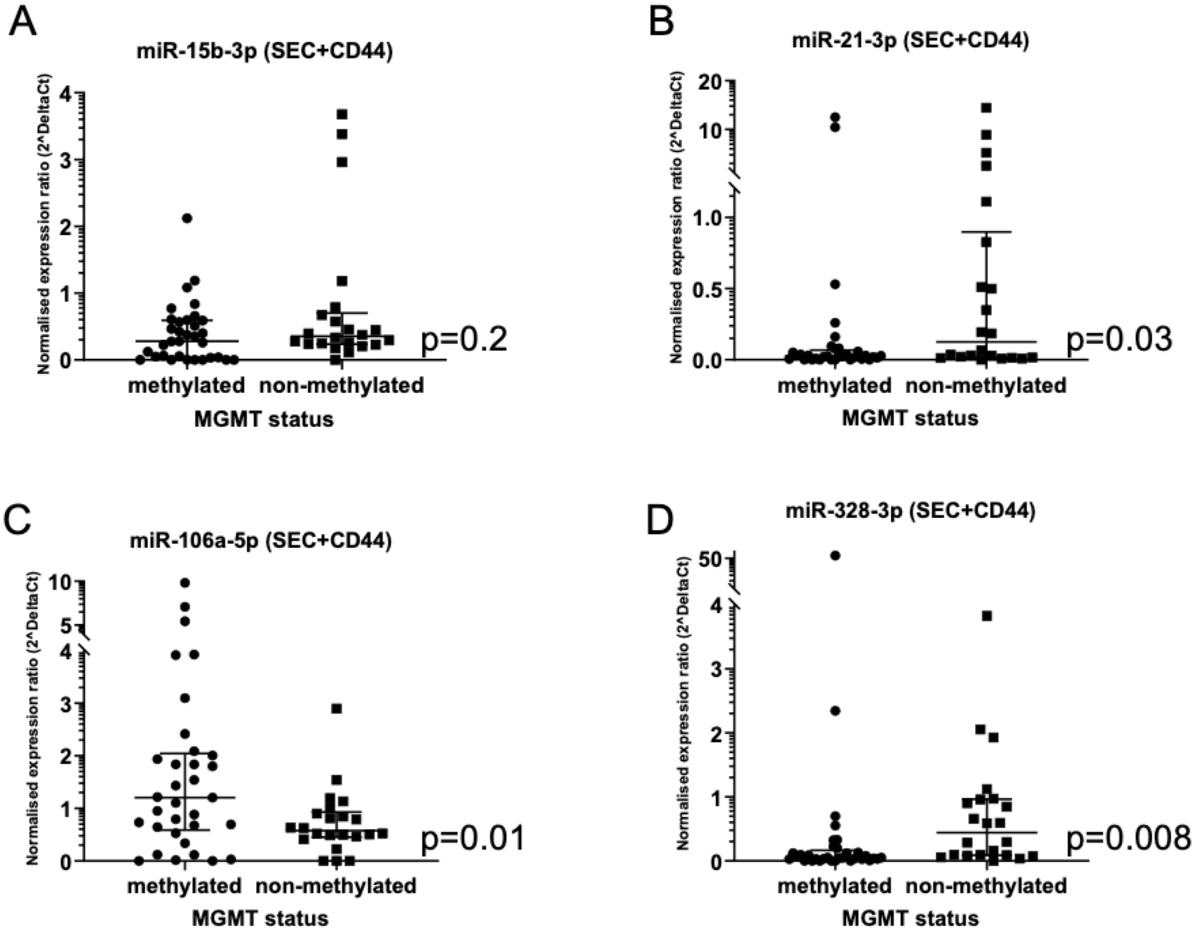

Figure S1: MicroRNA expression in SEC+CD44 EV depending on MGMT promoter methylation status.

Normalized expression ratio (2^deltaCt) of four microRNAs in SEC+CD44 EV from glioblastoma patients (n=55, with n=33 methylated and n=22 non-methylated). (A-D: miR-15b-3p, miR-21-3p, miR-106a-5p, miR-328-3p) Expression levels were compared using a Wilcoxon rank sum test.

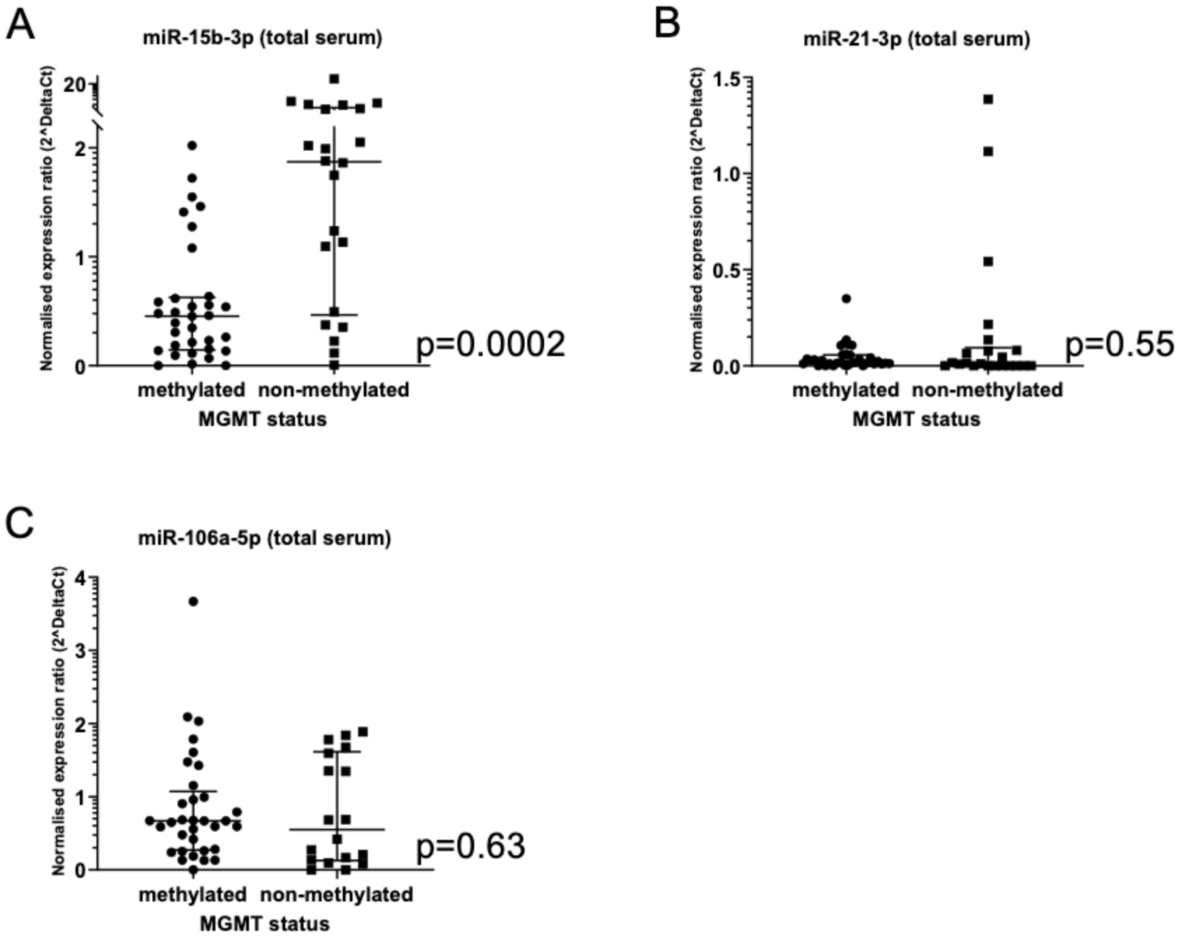

Figure S2: MicroRNA expression in total serum depending on MGMT promoter methylation status.

Normalized expression ratio (2^deltaCt) of four microRNAs in SEC+CD44 EV from glioblastoma patients (n=55, with n=33 methylated and n=22 non-methylated). **(**A-C: miR-15b-3p, miR-21-3p, miR-106a-5p) Expression levels were compared using a Wilcoxon rank sum test.

Patient characteristics	
n	55
Age (years)	56 (19-77)
Sex	
Women	24 (44%)
Men	31 (56%)
Median overall survival (years)	2.35 (0.39-5.17)
IDH mutation status	
IDH-wildtype	53 (96%)
IDH-mutated	2 (4%)
Unknown	0 (0%)
MGMT promoter methylation	
MGMT-methylated	33 (60%)
Non-MGMT-methylated	22 (40%)

Table S1: Patient characteristics.

miRNA ID	Assay ID	Mature miRNA sequence	Attribute
miR-103a-3p	478253_mir	AGCAGCAUUGUACAGGGCUAUGA	Reference
miR-484	478308_mir	UCAGGCUCAGUCCCCUCCCGAU	
let-7a-5p	478575_mir	UGAGGUAGUAGGUUGUAUAGUU	GB Marker
miR-10b-5p	478494_mir	UACCCUGUAGAACCGAAUUUGUG	
miR-15b-3p	477929_mir	CGAAUCAUUAUUUGCUGCUCUA	
miR-21-3p	477973_mir	CAACACCAGUCGAUGGGCUGU	
miR-23a-3p	478532_mir	AUCACAUUGCCAGGGAUUUCC	
miR-106a-5p	478225_mir	AAAAGUGCUUACAGUGCAGGUAG	
miR-124-3p	003188_mat	UAAGGCACGCGGUGAAUGCCAA	
miR-125a-5p	477884_mir	UCCCUGAGACCCUUUAACCUGUGA	
miR-128-3p	477892_mir	UCACAGUGAACCGGUCUCUUU	
miR-133a-5p	478706_mir	AGCUGGUAAAAUGGAACCAAAU	
miR-155-5p	483064_mir	UUAAUGCUAAUCGUGAUAGGGGUU	
miR-182-5p	477935_mir	UUUGGCAAUGGUAGAACUCACACU	
miR-199a-3p	477961_mir	ACAGUAGUCUGCACAUUGGUUA	
miR-328-3p	478028_mir	CUGGCCCUCUCUGCCCUUCCGU	
miR-486-5p	478128_mir	UCCUGUACUGAGCUGCCCCGAG	

Table S2: List of microRNAs (miR ID)

List of microRNAs (miR ID) including the Thermo Fisher assay number (Assay ID) and mature microRNA sequence analysed in this study either as reference-microRNAs or as glioblastoma (GB) markers (Attribute).

Single Analysis	miRNA	P value	Rank	FDR q=10% * (i=Rank / m tests=28)	significant Benjamini-Hochberg
SEC+CD44	miR-15b-3p ↑	0.28	18	0.06	
	miR-21-3p ↑	0.04	9	0.03	
	miR-328-3p ↑	0.26	15	0.05	
	miR-106a-5p ↓	0.09	13	0.05	
total serum (t.s.)	miR-15b-3p ↑	0.013	7	0.03	*
	miR-21-3p ↑	0.86	27	0.1	
	miR-328-3p ↑	n.c.	-	-	
	miR-106a-5p ↓	0.59	26	0.09	
**Combined Analysis**					
total serum (t.s.) + SEC+CD44	miR-15b-3p(t.s.) ↑ + miR-21-3p (SEC+CD44) ↑	0.003	2	0.007	*
	miR-15b-3p (t.s.) ↑ + miR-106a-5p (SEC+CD44) ↓	0.002	1	0.004	*
	miR-15b-3p(t.s.) ↑ + miR-328-3p (SEC+CD44) ↑	0.003	3	0.01	*
SEC+CD44	miR-15b-3p↓+miR-106a-5p↓	0.024	8	0.03	*
	miR-106a-5p↓+miR-21-3p↑	0.007	6	0.02	*
	miR-106a-5p↓+miR-328-3p↑	0.005	4	0.01	*

Table S3: Rank of log-rank p values of different dichotomous microRNA survival analyses

Rank of log-rank p values of different dichotomous microRNA survival analyses (miR-15b-3p, miR-21-3p, miR-106a-5p and miR-328-3p) either alone or in combination with SEC+CD44 separated EVs (labelled SEC+CD44) or total serum (labelled t.s.) from glioblastoma patients. Depicted are the markers, the log-rank p values and the Benjamini-Hochberg critical value calculated as outlined in the statistical section. Green highlighting indicates significance after Benjamini-Hochberg correction for multiple testing.

Marker 1	Marker 2	p-value	Rank	q(FDR=10%) * (i/m)	significant Benjamini Hochberg
miR-15 t.s.	miR-106 SEC44	0.002	1	0.004	*
miR-15 t.s.	miR-21 SEC44	0.003	2	0.007	*
miR-15 t.s.	miR-328 SEC44	0.003	3	0.01	*
miR-106 SEC44	miR-328 SEC44	0.005	4	0.01	*
miR-15 t.s.	miR-15 SEC44	0.005	5	0.02	*
miR-21 SEC44	miR-106 SEC44	0.007	6	0.02	*
miR-15 t.s.	-	0.01	7	0.03	*
miR-15 SEC44	miR-106 SEC44	0.02	8	0.03	*
miR-21 SEC44	-	0.04	9	0.03	
miR-15 t.s.	miR-106 t.s.	0.05	10	0.04	
miR-21 SEC44	miR-328 SEC44	0.07	11	0.04	
miR-106 t.s.	miR-106 SEC44	0.08	12	0.04	
miR-106 SEC44	-	0.09	13	0.05	
miR-15 t.s.	miR-21 t.s.	0.19	14	0.05	
miR-328 SEC44	-	0.26	15	0.05	
miR-15 SEC44	miR-21 SEC44	0.28	16	0.06	
miR-21 t.s.	miR-15 SEC44	0.28	17	0.06	
miR-15 SEC44	-	0.28	18	0.06	
miR-21 t.s.	miR-106 SEC44	0.29	19	0.07	
miR-21 t.s.	miR-106 t.s.	0.32	20	0.07	
miR-106 t.s.	miR-21 SEC44	0.36	21	0.08	
miR-21 t.s.	miR-21 SEC44	0.43	22	0.08	
miR-106 t.s.	miR-328 SEC44	0.43	23	0.08	
miR-106 t.s.	-	0.59	24	0.09	
miR-21 t.s.	miR-328 SEC44	0.60	25	0.09	
miR-106 t.s.	miR-15 SEC44	0.76	26	0.09	
miR-21 t.s.	-	0.86	27	0.10	
miR-15 SEC44	miR-328 SEC44	0.94	28	0.10	

Table S4: Rank of log-rank p values of different dichotomous microRNA survival analyses showing all correlations

Rank of log-rank p values of different dichotomous microRNA survival analyses showing all correlations (miR-15b-3p, miR-21-3p, miR-106a-5p and miR-328-3p) either alone or in combination with SEC+CD44 separated EVs (labelled SEC+CD44) or total serum (labelled t.s.) from glioblastoma patients. Depicted are the markers, the log-rank p values and the Benjamini-Hochberg critical value, calculated as outlined in the statistical section. Green highlighting indicates significance after Benjamini-Hochberg correction for multiple testing.
